# Actin-dependent mitochondrial internalization in cardiomyocytes: evidence for rescue of mitochondrial function

**DOI:** 10.1242/bio.201511478

**Published:** 2015-04-10

**Authors:** Christina A. Pacak, Janine M. Preble, Hiroshi Kondo, Peter Seibel, Sidney Levitsky, Pedro J. del Nido, Douglas B. Cowan, James D. McCully

**Affiliations:** 1 Department of Pediatrics, University of Florida College of Medicine, Gainesville, FL 32607, USA; 2 Division of Cardiac Surgery, Beth Israel Deaconess Medical Center, Boston, MA 02215, USA; 3 Universitat Leipzig, Molekulare Zelltherapie, Biotechnologisch-Biomedizinisches Zentrum, 04103 Leipzig, Germany; 4 Harvard Medical School, Boston, MA 02115, USA; 5 Division of Cardiac Surgery, Boston Children's Hospital, Boston, MA 02115, USA; 6 Department of Anesthesiology, Perioperative and Pain Medicine, Boston Children's Hospital, Boston, MA 02115, USA

**Keywords:** Mitochondria, Transplantation, Cardioprotection, Mitochondrial DNA, Endocytosis

## Abstract

Previously, we have demonstrated that the transplantation of viable, structurally intact, respiration competent mitochondria into the ischemic myocardium during early reperfusion significantly enhanced cardioprotection by decreasing myocellular damage and enhancing functional recovery. Our *in vitro* and *in vivo* studies established that autologous mitochondria are internalized into cardiomyocytes following transplantation; however, the mechanism(s) modulating internalization of these organelles were unknown. Here, we show that internalization of mitochondria occurs through actin-dependent endocytosis and rescues cell function by increasing ATP content and oxygen consumption rates. We also show that internalized mitochondria replace depleted mitochondrial (mt)DNA. These results describe the mechanism for internalization of mitochondria within host cells and provide a basis for novel therapeutic interventions allowing for the rescue and replacement of damaged or impaired mitochondria.

## INTRODUCTION

Myocardial ischemia/reperfusion injury is associated with mitochondrial damage and dysfunction that detrimentally alters oxygen consumption and energy synthesis (Lesnefsky et al., 2004; McCully et al., 2007). These events occur during ischemia and extend into reperfusion to severely compromise myocardial functional recovery and cellular viability. In previous studies, we demonstrated that the transplantation of viable, structurally intact, respiration competent, mitochondria, into the ischemic zone of the myocardium during early reperfusion significantly decreases myocardial ischemia/reperfusion injury (McCully et al., 2009; Masuzawa et al., 2013). These studies also established that transplanted mitochondria are internalized into cardiomyocytes and increase oxygen consumption rates and ATP production (Masuzawa et al., 2013).

The cellular mechanism(s) that enable the internalization of transplanted mitochondria were unknown. In this report, we investigate the potential mechanisms modulating mitochondrial internalization into cardiomyocytes using pharmacological blockers of clathrin mediated endocytosis, actin mediated endocytosis, macro-pinocytosis, and tunneling nanotubes (Bereiter-Hahn et al., 2008; Cowan et al., 2001; Huang et al., 2013; Islam et al., 2012; Kitani et al., 2014; Le et al., 2000; Lou et al., 2012; Mahammad and Parmryd, 2015; Masters et al., 2013; Nakase et al., 2004; Spees et al., 2006). We also investigate the effects of mitochondrial transplantation on the rescue of cell function and replacement of mtDNA in host cells with depleted mtDNA. Our results indicate that transplanted mitochondria are internalized by an actin dependent pathway. Once internalized the mitochondria rescue cell function and replace damaged mtDNA in host cells.

## RESULTS AND DISCUSSION

### Mitochondrial internalization into cardiomyocytes

Previously, we have demonstrated the uptake of autologous mitochondria *in vitro* and *in vivo* (Masuzawa et al., 2013). Our results demonstrated that transplanted mitochondria were internalized in host cardiomyocytes. In this report, we have extended these earlier studies using the pH sensitive pHrodo Red SE label as an indicator of endocytosis (Miksa et al., 2009; Neaga et al., 2013). pHrodo Red SE label enables fluorescent measurement of internalization based on the increase in fluorescence as the mitochondria are internalized within the cells (Miksa et al., 2009; Neaga et al., 2013).

**Fig. 1.**
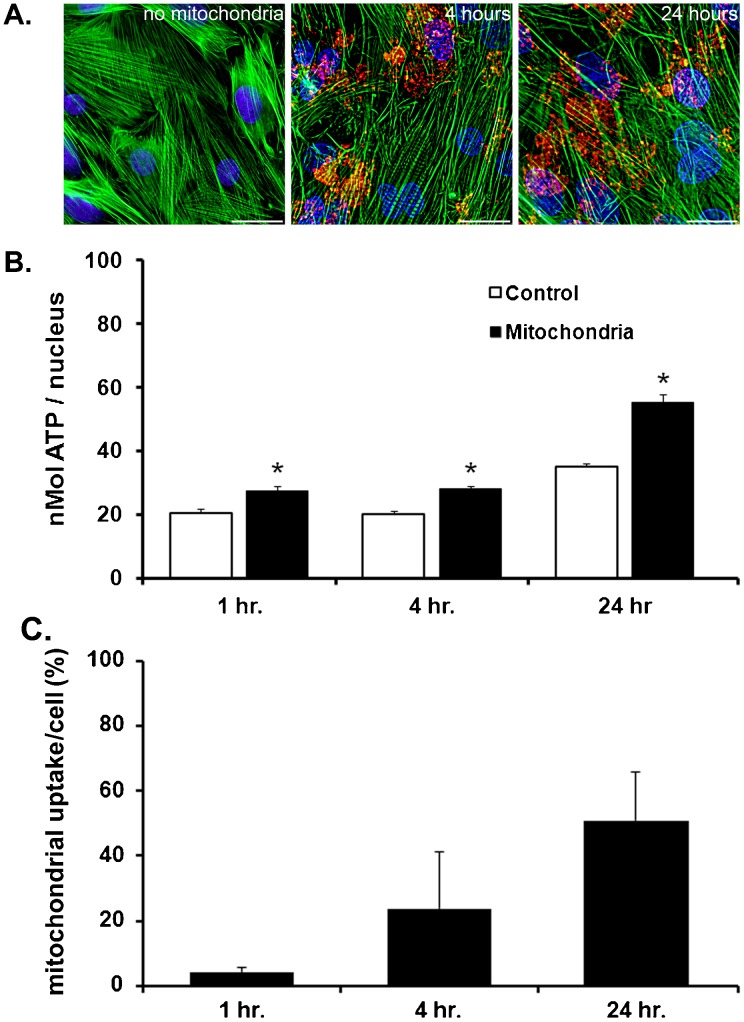
Mitochondrial internalization in cardiomyocytes. (A) Representative florescent micrographs of isolated rat liver mitochondria labeled with pHrodo co-incubated with cardiomyocytes for 4 hour (middle panel) or 24 hours (right panel). Control cardiomyocytes (no mitochondria) are shown in the left panel. In all images the blue stain is DAPI; red, pHrodo labeled mitochondria, green is 488 phalloidin (f-actin). Scale bars are 25 µm. Results show internalization of transplanted mitochondria at 4 and 24 hours. (B) ATP content in cardiomyocytes: ATP content (nmol/10^3^ cells) in control, no mitochondria (white bars) and cardiomyocytes co-incubated with mitochondria (black bars). (C) Mitochondrial uptake at 1, 4 and 24 hour co-incubation. The mean±sd for n = 5 experiments is shown. Results indicate that ATP content is significantly increased (p<0.05) in cardiomyocytes following 24 hours co-incubation with liver mitochondria. ATP standard luminescence; R^2^ = 0.9998. Statistical differences at p<0.05 vs. control is shown as *.

**Fig. 2.**
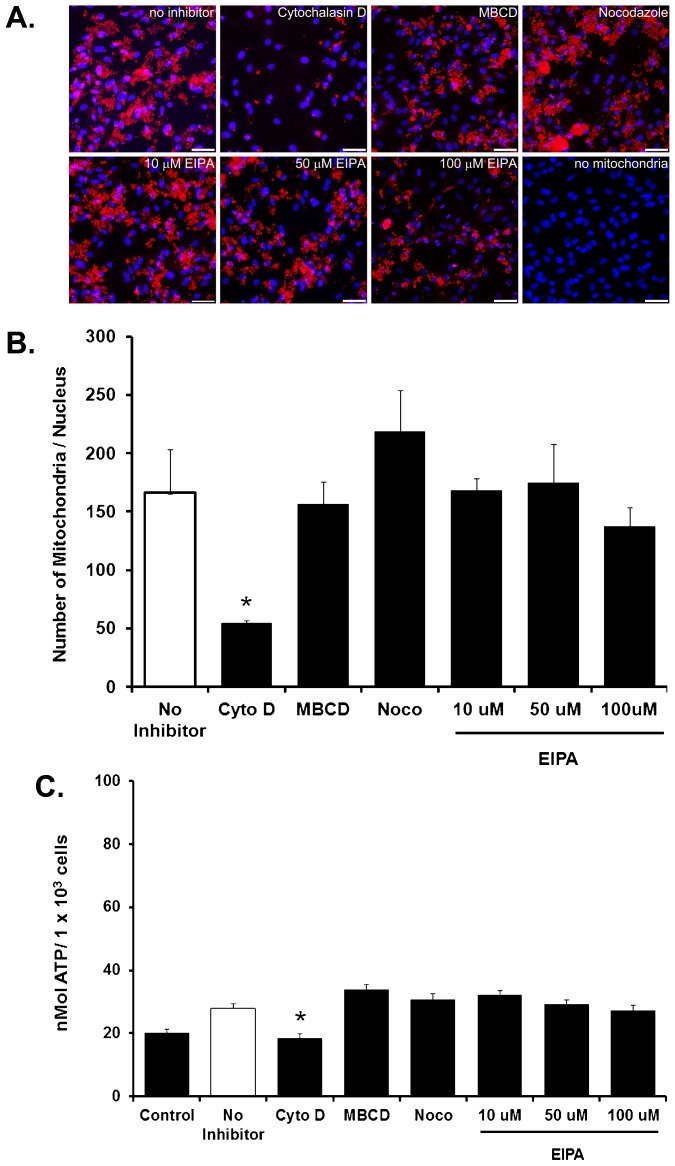
Inhibition of mitochondrial internalization. (A) Representative florescent micrographs of 2-day cardiomyocytes co-incubated with 1×10^7^ rat liver mitochondria labeled with pHrodo red following 4 hours co-incubation. Actin-dependent endocytosis and phagocytosis was inhibited with cytochalasin D (CytoD), caveola-dependent-clathrin dependent endocytosis was inhibited with methyl-β–cyclodextrin (MβCD), tunneling nano tube formation was inhibited with nocodazole (Noco) and macro pinocytosis was blocked with 5-(N-Ethyl-N-isopropyl)amiloride (EIPA) at 10 µM, 50 µM or 100 µM . Control cardiomyocytes were co-incubated with mitochondria without inhibitor (no inhibitor), As separate control, cardiomyocytes without mitochondria (no mitochondria) is also shown. Internalized mitochondria are red (pHrodo labeled mitochondria). Nuclei are shown in blue (DAPI). Scale bars are 25 µm. (B) Internalization of mitochondria per nucleus as determined by florescent microscopy following 4 hours of co-incubation. (C) ATP content (nmol/10^3^ cells). Results are shown as the mean and standard deviation for n = 5 in each group. Statistical differences at p<0.05 vs. cardiomyocytes co-incubated for 4 hours with mitochondria without inhibitor (no inhibitor) is shown as *. Results demonstrate that inhibition of actin-dependent endocytosis and phagocytosis inhibits the internalization of transplanted mitochondria and decreases ATP content.

Our results recapitulate our previous observations and demonstrate that cardiomyocytes co-incubated with mitochondria are internalized in a time-dependent manner. Mitochondrial internalization into cardiomyocytes is evident following 1 hour co-incubation (26±8.2 mitochondrial per nucleus) and is increased following 4 hours co-incubation (186±24.6 mitochondrial per nucleus) and following 24 hours co-incubation (257±69.1 mitochondrial per nucleus; Fig. 1A). The percentage of mitochondria internalized at each time point is shown in Fig. 1C. based on binucleated cardiomyocyte population (Stephen et al., 2009). The internalized mitochondria significantly increase (p<0.05) cardiomyocyte ATP content as compared to control (Fig. 1B) in agreement with our previous results (Masuzawa et al., 2013). The potential mechanisms by which mitochondria could be internalized by cardiomyocytes were investigated using specific blockers based on their wide use and established selectivity (Ivanov, 2008). Cytochalasin D (CytoD, 10 µM) was used to block actin polymerization (Bereiter-Hahn et al., 2008; Cowan et al., 2001), methyl-β-cyclodextrin (MβCD, 1 mM) was used to block caveola-dependent-clathrin dependent endocytosis (Le et al., 2000; Mahammad and Parmryd, 2015; Pfeiffer et al., 2014) nocodazole (Noco, 4 ng/mL) was used to block tunneling nano tubes (Huang et al., 2013; Islam et al., 2012; Lou et al., 2012; Spees et al., 2006), and 5-(N-Ethyl-N-isopropyl)amiloride (EIPA) at 10 µM, 50 µM or 100 µM was used to block macro-pinocytosis (Kitani et al., 2014; Nakase et al., 2004) (Fig. 2A,B).

Our studies show that mitochondrial internalization by cardiomyocytes following 4 hours co-incubation is unaffected following pre-incubation with methyl-β-cyclodextrin, nocodazole, or with 5-(N-Ethyl-N-isopropyl)amiloride suggesting that tunneling nanotubes, caveola-dependent-clathrin dependent endocytosis and macro-pinocytosis are not involved in mitochondrial internalization into cardiomyocytes (Fig. 2A–C). Only pre-incubation with cytochalasin D significantly decreased (p<0.05) the internalization of mitochondria into cardiomyocytes and decreased ATP content as compared to no inhibitor (Fig. 2A–C).

The ability of cytochalasin D to inhibit actin-dependent endocytosis and phagocytosis has been well characterized and this mechanism would agree with the proposed endosymbiosis origin of mitochondria (Ivanov, 2008; Margulis, 1975). Other mechanisms are less likely to be involved based on the method of mitochondrial presentation and mitochondrial size. In all of our studies, mitochondria are presented externally and subsequently become internalized by host cells. This is in contrast to intercellular mitochondrial transfer where it has been proposed that mitochondria are passed from one individual cell to another via tunneling nanotubes (Huang et al., 2013; Islam et al., 2012; Lou et al., 2012; Spees et al., 2006). Although it is possible that intercellular mitochondrial transfer occurs following internalization, this mechanism does not modulate initial mitochondrial internalization.

The role of macro-pinocytosis while recently suggested to play a role in mitochondrial internalization (Kitani et al., 2014) also does not appear to be involved in mitochondrial internalization into cardiomyocytes. Kitani et al., 2014, used 25 or 50 µM EPIA to block mitochondrial internalization in human uterine endometrial cancer cells. In our studies we have used three concentrations (10, 50 and 100 µM) of EIPA and found no decrease in the internalization of mitochondria into cardiomyocytes suggesting that in cardiomyocytes, macro-pinocytosis does not play a role in mitochondrial internalization.

The mechanism by which the internalized mitochondria escape endosomes remains to be elucidated and is beyond the scope of this report. Our results agree with our previous observations where no cellular extensions in either cardiomyocytes (*in vitro*) or myocardial sections (following *in vivo* mitochondrial transplantation experiments) were detected and no colocalization of internalized mitochondria with any lysosomal, caveolae or autophagosomal markers was observed (Masuzawa et al., 2013). Overall, our results establish that internalization of mitochondria into cardiomyocytes is time dependent and occurs by actin-dependent endocytosis.

### Rescue of mitochondrial function and replacement of mtDNA

To characterize the functional contribution of internalized mitochondria, we performed studies using HeLa p^0^ cells depleted of mtDNA (Kukat et al., 2008). HeLa p^0^ cells are capable of energy generation through fermentation but lack oxygen consumption capacity due to depletion of electron transport chain proteins encoded by mtDNA. Our studies demonstrate that co-incubation of HeLa p^0^ cells with mitochondria isolated from HeLa cells containing intact mtDNA rescues HeLa p^0^ cell function by significantly increasing ATP content and oxygen consumption rates.

**Fig. 3.**
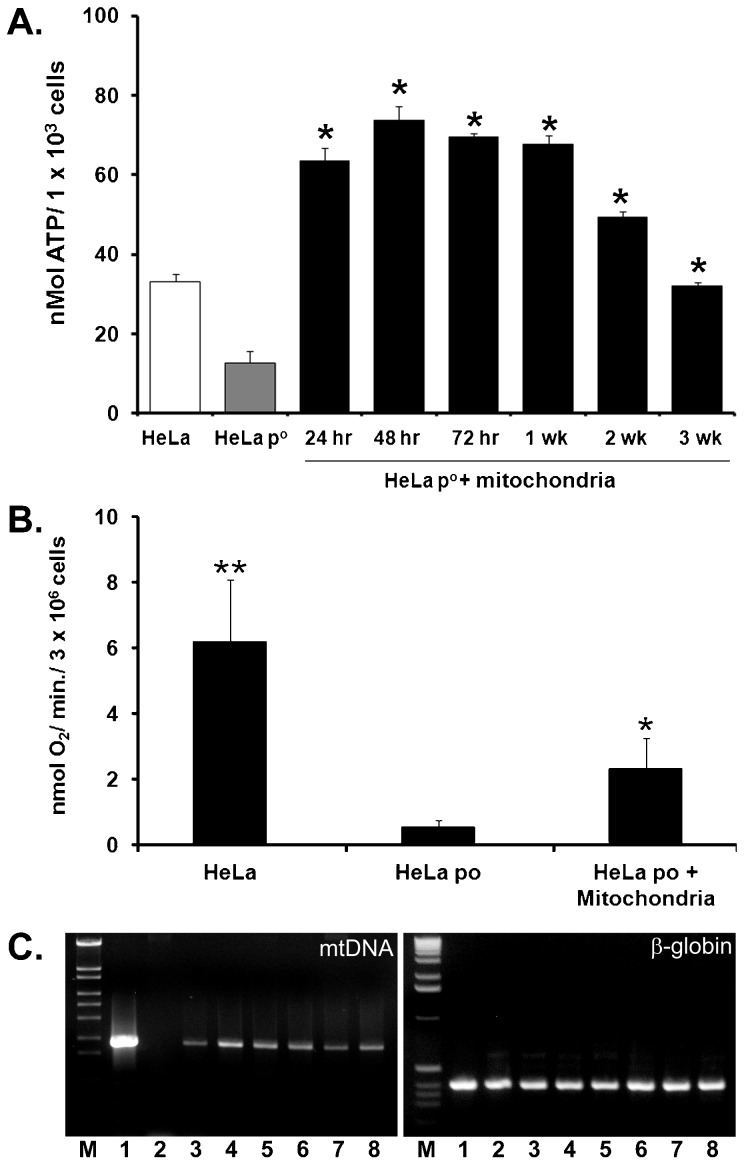
Rescue of HeLa p^0^ cell function following mitochondrial transplantation. HeLa p^0^ cells depleted of mitochondrial DNA were co-incubated with mitochondrial from HeLa cells containing intact mitochondrial DNA for 3 weeks. Control HeLa and HeLa p^0^ cells were incubated with vehicle only. (A) ATP content (nmol ATP/1×10^3^ cells). ATP content in HeLa p^0^ cells was significantly increased following mitochondrial internalization. Results are shown as the mean and standard deviation for n = 8–10 in each group. Statistical differences at p<0.05 vs. HeLa p^0^ are shown as *. (B) Oxygen consumption rate. The oxygen consumption rate (nmol/min/3×10^6^ cells) at 3 weeks culture is shown for HeLa, HeLa p^0^, and HeLa p^0^ cells co-incubated with HeLa mitochondria. Oxygen consumption rate was significantly increased in Hela p^0^ cells co-incubated with HeLa mitochondria. Results are shown as the mean±sd for n = 8–10 in each group. Statistical differences at p<0.05 vs. HeLa p^0^ are shown as *. Statistical differences at p<0.5 vs HeLa p^0^ and HeLa p^0^ cells co-incubated with HeLa mitochondria are shown as **. (C) Representative agarose gels of PCR analysis products in HeLa, HeLa p^0^, and HeLa p^0^ cells co-incubated with HeLa mitochondria. PCR products for mtDNA (left panel, 1760 bp) and PCR products for nuclear DNA (Human beta-globin, control, right panel, 480 bp) are shown. Molecular size markers are shown (M). Lanes are 1: HeLa, 2, HeLa p^0^. Lanes 3–8 are HeLa p^0^ cells co-incubated with HeLa mitochondria at 3: 24 hours, 4: 48 hours, 5: 72 hours. 6: 1 week, 7: 2 weeks and 8: 3 weeks co-culture. mtDNA was not detected in HeLa p^0^ cells. mtDNA was observed in HeLa and in HeLa p^0^ cells co-incubated with HeLa mitochondria at 24 hours and remained detectable for at least 3 weeks following co-culture. All results represent an n = 4 for each group.

ATP content was significantly increased in HeLa p^0^ cells following co-incubation with mitochondria at 24, 48, 72 hours and 1 and 2 weeks. The enhanced intracellular ATP content corresponds to significant increases in oxygen consumption rates of HeLa p^0^ cells after mitochondrial internalization (Fig. 3B).

The effects of mitochondrial transplantation on the rescue of mitochondrial function in HeLa p^0^ cells were not absolute as ATP content and oxygen consumption rate were significantly decreased as compared to that in HeLa cells (Fig. 3A,B). This is likely due to the culture conditions used in our studies that required media containing high glucose (4.5 g/l D-glucose) and 50 mg/ml uridine (Hashiguchi and Zhang-Akiyama, 2009). We observed that HeLa cells grown in this medium grew more slowly than those grown with 1 g/l D-glucose and no uridine (results not shown). Thus, the number of control HeLa p0 cells as compared to HeLa p0 cells containing internalized HeLa mitochondria may have been disproportionally overrepresented due to the medium being optimized specifically for their growth. We did not change the media to low glucose media following co-incubation with mitochondria as this would have limited the survival of HeLa p^0^ cells that did not internalize mitochondria from HeLa cells HeLa p^0^ cells cannot survive in low glucose media due to the complete reliance on glucose fermentation for ATP synthesis. HeLa p^0^ cells also require media containing uridine due to a deficiency of pyrimidine biosynthesis (Hashiguchi and Zhang-Akiyama, 2009).

Of significance, PCR analysis clearly demonstrated replacement of mtDNA in HeLa p^0^ cells following mitochondrial transplantation. We were unable to quantify the absolute levels of mtDNA in the rescued HeLa p^0^ cells as HeLa cells contain an unbalanced number of chromosomes (Adey et al., 2013). While our results show that the absolute quantity of mtDNA in HeLa p0 cells co-incubated with Hela cell mitochondria (containing intact mtDNA) is significantly less than that observed in HeLa cells, the mtDNA present is sufficient to significantly enhance intracellular ATP content and oxygen consumption rate as compared to untreated HeLa p0 cells. We did not investigate if the mtDNA in the mitochondria transplanted into HeLa p^0^ cells replicated or if the transplanted mitochondrial population expanded as the rescued HeLa cells divided as this was beyond the scope of the current paper and thus remain to be demonstrated.

Although the rescue of cardiomyocytes following ischemia and reperfusion injury is the primary goal of our research, the potential of mitochondrial transplantation is not limited to cardioprotection alone. Our methodology has recently been utilized by others to show protection from ischemia-reperfusion injury in the liver (Lin et al., 2013) and to enhance drug sensitivity in human cancer cells (Elliott et al., 2012). In summary, our data suggest that mitochondrial transplantation has potential to rescue cell function and replace damaged mitochondrial DNA in many cell types and disease states.

In conclusion, we show that the internalization of mitochondria in cardiomyocytes occurs through actin-dependent endocytosis and rescues cell function through enhanced ATP content and oxygen consumption rate. We also show that internalized mitochondria replace depleted mtDNA. These results provide a mechanism for the internalization of mitochondria within host cells and a basis for novel therapeutic interventions allowing for the rescue and replacement of damaged or impaired mitochondria.

## MATERIALS AND METHODS

### Cardiomyocytes isolation and culture

Neonatal rat cardiomyocytes were isolated from 1-day-old Lewis rat pups the Neonatal Cardiomyocyte Isolation System (Worthington Biochemicals, Lakewood, NJ, USA (Masuzawa et al., 2013).

### Mitochondrial isolation and labeling

Mitochondria were isolated and viability was determined as previously described (Masuzawa et al., 2013). The isolated mitochondria were labeled with pHrodo red particle label (Life Technologies, Grand Island, NY, USA) (Miksa et al., 2009; Neaga et al., 2013) for 10 minutes at 4°C and then washed four times in respiration buffer (250 mmol/l sucrose, 2 mmol/l KH_2_PO_4_, 10 mmol/l MgCl_2_, 20 mmol/l K^+^-HEPES buffer, pH 7.2, 0.5 mmol/l K^+^-EGTA, pH 8.0, 5 mmol/l glutamate, 5 mmol/l malate, 8 mmol/l succinate, 1 mmol/l ADP). The labeled mitochondria were resuspended in fresh respiration buffer and the last wash supernatant was saved. The labeled mitochondrial (1×10^7^/well) were co-incubated with cardiomyocytes (50,000/well). At the conclusion of each time point, the media was removed and the cells were washed four times with 1× PBS and 200 µl of fresh medium was added to each well. Control cardiomyocytes were co-incubated with the last pHrodo wash. Mitochondrial internalization was determined using ImageJ 1.48 software (imagej.nih.gov/ij/download/).

### Blockers of mitochondrial internalization

Following two days of culture, cardiomyocytes were pre-treated for 30 minutes with either cytochalasin D (CytoD, 10 µM) (Bereiter-Hahn et al., 2008; Cowan et al., 2001; Kastl et al., 2013), methyl-β-cyclodextrin (MβCD, 1 mM) (Le et al., 2000; Mahammad and Parmryd, 2015; Pfeiffer et al., 2014), nocodazole (Noco, 4 ng/mL) (Huang et al., 2013; Islam et al., 2012; Lou et al., 2012; Spees et al., 2006) or 5-(N-Ethyl-N-isopropyl) amiloride (EIPA, 10 µM, 50 µM or 100 µM) (Kitani et al., 2014; Nakase et al., 2004) ; all from Sigma-Aldrich, St. Louis, MO, USA). All blockers were dissolved in dimethyl sulfoxide (DMSO, Sigma-Aldrich, St. Louis, MO, USA) and resuspended in media (≥1:10,000 dilution). Control cardiomyocytes were pre-treated by co-incubation with an equal molar concentration of DMSO in media for 30 minutes. Following pre-treatment, the media was removed and the cardiomyocytes were washed four times with 1× PBS, and 200 µl of fresh media was added to each well. The cardiomyocytes were then co-incubated with pHrodo labeled mitochondria (1×10^7^/well) or with the final pHrodo wash. Internalization of mitochondria was determined by as described above. All results were compared to control cardiomyocytes co-incubated with pHrodo-labeled mitochondria.

### ATP determination

ATP content was determined using the ATPlite Luminescence ATP Detection Assay System (Perkin Elmer, Waltham, MA, USA) (Diepart et al., 2010; Lanza and Nair, 2009; Manfredi et al., 2002). All assays were performed in the absence of fluorescent dyes as it has been previously reported that these dyes may interfere with mitochondrial function (Poot et al., 1996).

### Rescue of mitochondrial function and replacement of mitochondrial DNA in HeLa p^0^ cells

HeLa p^0^ cells containing deleted mtDNA (Kukat et al., 2008) were co-incubated with isolated mitochondria from HeLa cells containing intact mtDNA. mtDNA deletion in HeLa p^0^ cells was confirmed by PCR analysis and by oxygen consumption rate. HeLa cells were cultured as described (Masuzawa et al., 2013). HeLa p^0^ cells were cultured in DMEM-high glucose-GLUTAmax (4.5 g/l) with 10% fetal bovine serum, 100 mg/ml sodium pyruvate, 50 mg/ml uridine and Antibiotic-Antimycotic (all from Life Technologies, Grand Island, NY, USA) (Hashiguchi and Zhang-Akiyama, 2009; Kukat et al., 2008).

HeLa p^0^ cells were cultured in 24 well plates (5,000/well) for 24 hours. Mitochondria were isolated from HeLa cells and co-incubated (1×10^7^/well) with HeLa p^0^. Control HeLa p^0^ cells were co-incubated with vehicle (respiration buffer) only. Following 72 hours, the cells were washed four times in 1× PBS, collected by trypsinization and then replated on 100 mm culture plates for one, two and three weeks. Cell cultures were split at 80% confluence. At specified end points, the cells were washed four times in 1× PBS and then collected by trypsinization. The cells were then used for determination of ATP content, mtDNA and oxygen consumption rate.

### Oxygen consumption rate

Oxygen consumption rate was determined by Oxytherm (Hansatech Instruments Ltd, Norfolk, UK) (Papandreou et al., 2006). In brief, 3×10^6^ cells were suspended in fresh DMEM-high glucose-GLUTAmax with 10% fetal bovine serum, 100 mg/ml sodium pyruvate, 50 mg/ml uridine and Antibiotic-Antimyctic (all from Life Technologies, Grand Island, NY, USA) at 37°C. The cell suspension was transferred to the oxytherm chamber and oxygen consumption was determined for 5 minutes and then cyanide (0.2 M) was added to terminate the respiration.

### PCR analysis

DNA was isolated and PCR was performed as described (Levitsky et al., 2003). The primers used for mtDNA detection were (5′-CAAATATCATTCTGAGG-3′) and (5′-GTTTTTGGGGTTTGGCA-3′) (Levitsky et al., 2003). The human B-globin gene was used as a control; GH20 (5′-GAAGAGCCAAGGACAGGTAC-3′) and GH21 (5′-GGAAAATAGACCAATAGGCAG-3′) (TaKaRa Shuzo Co. Ltd, Otsu, Japan).

### Fluorescent microscopy

Fluorescent microscopy studies were performed as previously described (Masuzawa et al., 2013). Some samples were stained with MitoTracker Red CMX Ros (100 nmol/l), Alexa 488-phalloidin (1:50 dilution), and DAPI (500 nmol/l) (all from Invitrogen, Grand Island, NY, USA). Primary antibodies were detected with highly cross-absorbed goat anti-mouse or anti-rabbit Alexa-conjugated secondary antibodies (Invitrogen, Grand Island, NY, USA).

### Statistical analysis

Statistical analysis was performed using SAS (version 6.12) software package (SAS Institute, Cary, NC, USA). The mean±SE for all data was calculated for all variables. Statistical significance was assessed using repeated measures analysis of variance (ANOVA) with group as a between subjects factor and time as a within subjects factor. Tukey honestly significant difference test was used for comparisons between control and other groups to adjust for the multiplicity of tests. Statistical significance was claimed at P<0.05.
